# Comparison of the Effect of Inhalation Aromatherapy with 10% and 30% Peppermint Essential Oils on the Severity of Nausea in Abdominal Surgery Patients

**DOI:** 10.1155/2020/5897465

**Published:** 2020-04-20

**Authors:** Yasin Ahmadi, Jahangir Rezaei, Mansour Rezaei, Alireza Khatony

**Affiliations:** ^1^Student Research Committee, Kermanshah University of Medical Sciences, Kermanshah, Iran; ^2^Clinical Research Development Center, Imam Reza Hospital, Kermanshah University of Medical Sciences, Kermanshah, Iran; ^3^Health Institute, Social Development and Health Promotion Research Center, Kermanshah University of Medical Sciences, Kermanshah, Iran; ^4^Health Institute, Social Development and Health Promotion Research Center, Kermanshah University of Medical Sciences, Kermanshah, Iran; ^5^Clinical Research Development Center, Imam Reza Hospital, Kermanshah University of Medical Sciences, Kermanshah, Iran

## Abstract

**Background:**

One of the most common surgical complications is nausea. Regarding the contradictory findings on the effect of aromatherapy with peppermint on the severity of nausea, in the present study, we aimed at comparing the effect of aromatherapy with 10% and 30% peppermint essential oils on the severity of nausea in surgical patients.

**Methods:**

This single-blind randomized controlled trial was conducted at the surgical ward of Imam Reza Hospital in Kermanshah, Iran. A total of 120 patients undergoing abdominal surgery were randomly divided into three groups of 10% peppermint, 30% peppermint, and control (40 patients in each group) using a random number table. In each of the intervention groups, 0.2 ml of 10% and 30% peppermint essential oil was inhaled. In the control group, the same amount of distilled water colored with green food coloring was inhaled. The severity of nausea was measured by nausea visual analog scale (NVAS) before and 10 minutes after the intervention.

**Results:**

In the 10% peppermint group, the mean severity of nausea before the intervention was 52.3 ± 13.7 out of 100, which reduced to 40.5 ± 13.5 after the intervention (*p* < 0.001). In the 30% peppermint group, the mean severity scores of nausea before and after the intervention were 60.2 ± 15.0 and 39.7 ± 12.4, respectively (*p* < 0.001). In the control group, the mean severity scores of nausea before and after the intervention was not statistically significant. There was no significant difference between the two intervention groups in terms of the mean severity of nausea after the intervention.

**Conclusions:**

It can be concluded that 10% and 30% peppermint essential oils are equally effective on the severity of nausea.

## 1. Introduction

Nausea is the most common postoperative complication and its most common causes are anesthesia, type of surgery, anxiety, stress, and type of anesthesia [1, 2]. Approximately 30% to 37% of surgical patients and 40% to 77% of abdominal surgery patients experience postoperative nausea [3, 4]. Postoperative nausea and vomiting can lead to outcomes such as pulmonary aspiration, rupture of surgical wounds, delayed healing, and dehydration [5, 6]. Pharmacological approaches are available for the prevention and treatment of postoperative nausea, including 5-HT3 receptor inhibitors (such as ondansetron), anti-inflammatory agents such as metoclopramide, and some antihistamines such as promethazine. Limited efficacy and side effects (e.g., drowsiness, headache, and confusion) affect the use of antinausea drugs [7, 8]. Evidence suggests that nonpharmacological methods, known as complementary therapies, are safer and have fewer side effects than medications [2, 9]. Aromatherapy is one of the types of complementary medicine that has recently attracted the attention of many researchers. In this method, a variety of herbal oils and essential oils are used [10, 11]. One of these aromatic oils is peppermint essential oil [12–14] which has mild side effects such as gastroesophageal reflux, allergic reactions, diarrhea, and heartburn [15]. Peppermint has over 1,000 different chemicals, the most effective of which are menthol (50%), menthone (16%), isomenthone (4%), and limonene (3%) [12–14]. Peppermint blocks the serotonin and dopamine receptors that are involved in nausea [16–19]. After inhalation, peppermint is absorbed through the lungs and nasotracheal mucosa and is rapidly absorbed by the bloodstream and exerts its rapid effects by acting on the limbic system [20].

The results of various studies have shown the anti-inflammatory properties of this plant. However, in some studies, peppermint has not been effective in reducing the severity of postoperative nausea [21, 22]. Some studies have used peppermint in combination with essential oils such as chamomile, orange, and fennel, as well as at the same concentration [23–25]. In some studies, the sample size was low [24, 26]. In this study, peppermint essential oil was used in two concentrations of 10% and 30% and the sample size was appropriate. Therefore, due to the limited number of studies evaluating the effect of peppermint inhalation on the severity of nausea in surgical patients and the inconsistent results of these studies, the present study aimed at comparing the effects of inhalation aromatherapy with 10% and 30% peppermint essential oils on the severity of nausea after abdominal surgery.

## 2. Materials and Methods

### 2.1. Study Design

This was a single-blind randomized controlled trial conducted from June 2014 to January 2015. The study was performed in the surgical ward of Imam Reza Hospital in Kermanshah, Iran.

### 2.2. Study Hypothesis

30% peppermint reduces the severity of postoperative nausea more than 10% peppermint.

### 2.3. Sample and Sampling Method

The study population consisted of all patients admitted to the surgical ward of Imam Reza Hospital, Kermanshah, for abdominal surgery. This hospital is the largest specialized center in western Iran which is located in Kermanshah city, west of Iran. The samples included patients admitted to the surgical ward who underwent surgery for reasons such as ileus, different types of cancer, and gallbladder diseases and met the inclusion criteria. Samples were selected by the convenience sampling method and randomly assigned to 10% peppermint, 30% peppermint, and control groups using a random number table. The inclusion criteria comprised of patient willingness, physician consent, nausea, NVAS score of 20, general anesthesia, good sense of smell (based on patient statements and researcher's examination), age 15 to 65 years, complete alertness, no respiratory diseases such as asthma, no allergic diseases, lack of consumption of antinausea or vomiting medications (intra- and postoperatively), no drug and smoking addiction, and lack of consumption of narcotics since 4 hours before the intervention. The exclusion criteria included patients with allergic symptoms such as cough and shortness of breath, administration of narcotics or antinausea and vomiting drugs (during the study), and death or transfer to another ward.

Ghani and Ibrahim's (2013) study was used to calculate the sample size [27]. Based on the formula for determining the ratio between two samples (*n*=(*z*_1−(*α*/2) _+*Z*_1−*β*_)^2^(*δ*_1_^2^+*δ*_2_^2^)/(*μ*_1_ − *μ*_2_)^2^), with 95% confidence and 90% test power, the standard sample size for each group was calculated at 34 individuals (102 subjects in total). To obtain more reliable results, 15% was added to the sample size and 40 individuals were included in each group (120 subjects in total).

### 2.4. Measurement Instrument

The tools used in this study included a demographic information form and nausea visual analogue scale (NVAS). The demographic information form included items on age, sex, type of surgery, length of time in the recovery room, and duration of anesthesia. NVAS is a visual scale that is divided from zero to one hundred. The validity and reliability of the NVAS have been reviewed and confirmed in the previous study [28]. In this instrument, zero is equivalent to the absence of nausea, and one hundred indicates the highest severity of nausea.

### 2.5. Interventions

**Figure 1:**
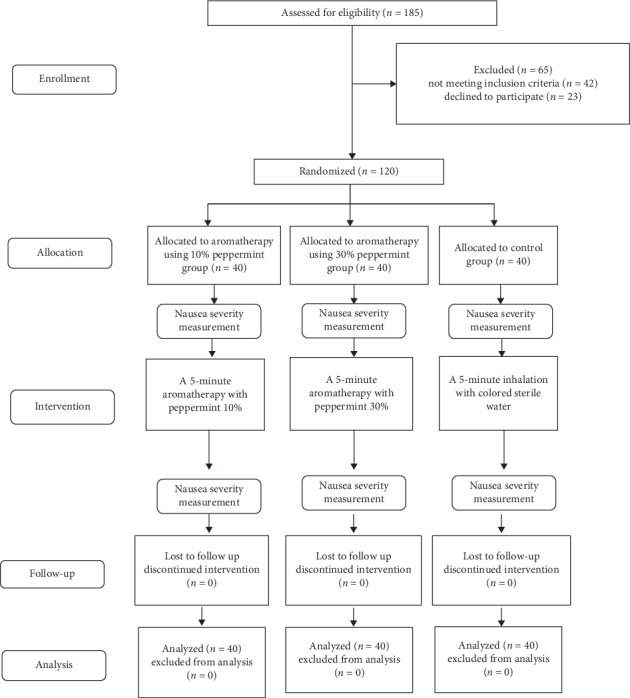
CONSORT flow diagram of the study.

After obtaining approval from the Ethics Committee of the university, the researcher started sampling, and those who met the inclusion criteria were randomly assigned to the three groups of 10% peppermint, 30% peppermint, and control (40 patients in each group) using a random number table. Before the intervention, in all three groups, the demographics form and NVAS were completed. Then, in the 10% peppermint group, 0.2 ml equivalent of two drops of 10% peppermint essential oil was added to 2 cc distilled water and poured onto a 4^*∗*^4 piece of gauze and placed at a 10 cm distance from the patient's nose for five minutes and the patient was asked to breathe normally. The same method was used in the 30% peppermint group, while 30% peppermint essential oil was used. In the control group, the same method was used and 2 cc distilled water colored with green food coloring was used as placebo. Also, to ensure the blindness of the study subjects, the distance between the beds of the intervention and control groups was more than two meters. Inhalation aromatherapy was performed only once for 5 minutes, according to the studies by Briggs et al. (2016) and Ferruggiari et al. (2012) [18, 21]. After the intervention, NVAS was again completed by the samples. It should be noted that all interventions were performed by the first author. The peppermint essential oils were produced by Shafa Medicinal Plants Company (Kurdistan, Iran) and had 100% purity. The containers of the peppermint essential oils and distilled water were quite similar and kept away from light in a refrigerator. The intervention was administered between 9 am and 12 pm (Figure 1).

### 2.6. Data Analysis

Data were analyzed by the Statistical Package for the Social Sciences (SPSS V.16.0; SPSS Inc., Chicago, IL, USA). The Kolmogorov–Smirnov test was used to test the hypothesis of normality of the data. The results indicated that the distribution of variables was normal. One-way analysis of variance (ANOVA) and Tukey's post hoc tests were used to compare the mean severity of nausea between the study groups before and after the intervention. Tukey's test was used to compare the severity of nausea before and after the intervention in a pairwise manner between the two groups. Paired *t*-test was used to compare the mean severity of nausea in each group before and after the intervention. The significant level was set at less than 0.05.

### 2.7. Ethical Considerations

The study was approved by the University Ethics Committee with the code of ethics 1691. The study was registered at the Iranian Registry of Clinical Trials with the code of IRCT20140414172567N1. The objectives of the study were explained to the participants, and their questions were answered. Written informed consent from the patients and physician consent were obtained. Samples were assured of the confidentiality of the information and their responses, and the samples were allowed to withdraw from the study at any time.

## 3. Results

In the present study, the mean age of the samples was 46.4 ± 12.1 years, and the most common surgical procedure was laparoscopic cholecystectomy (47.5%; *n* = 57). All the three study groups were homogeneous in terms of the demographic variables (Table 1).

In each of the intervention groups, there was a significant difference in the mean NVAS scores before and after aromatherapy (*p* < 0.001), but this difference was not significant for the control group. In the 10% peppermint group, the mean severity of nausea before the intervention was 52.3 ± 13.7 out of 100, which reduced to 40.5 ± 13.5 after the intervention (*p* < 0.001). In the 30% peppermint group, the mean severity scores of nausea before and after the intervention were 60.2 ± 15 and 39.7 ± 12.4, respectively (*p* < 0.001). In the control group, the mean severity scores of nausea before and after the intervention were 51 ± 17.4 and 47.8 ± 13.7, respectively, which was not statistically significant (Table 2). There was a statistically significant difference in the mean severity of nausea among the three groups before the intervention (*p* < 0.015). In this regard, the results of Tukey's post hoc test showed a significant difference between the control and 30% peppermint groups (*p* < 0.018). However, there was no statistically significant difference between the 10% and 30% peppermint groups and between the control and 10% peppermint groups. There was a significant difference between the study groups in the mean severity of nausea after the intervention (*p* < 0.001) (Table 2). In this regard, the results of Tukey's post hoc test showed a significant difference between the control and 10% peppermint groups and between the control and 30% peppermint groups (*p*=0.002). However, there was no statistically significant difference between the 10% and 30% peppermint groups (Table 3).

## 4. Discussion

Our objective was to compare the effect of inhalation aromatherapy with 10% and 30% peppermint essential oils on the severity of nausea in abdominal surgery patients. The results showed that the mean severity of nausea had equally reduced after the intervention in both groups of 10% and 30% peppermint as compared to preintervention. In the control group, the mean severity of nausea before and after the intervention did not change significantly. In this regard, during a clinical trial, Hunt et al. (2013) investigated the impact of inhalation aromatherapy on the severity of nausea in 301 patients after outpatient surgery. The results showed that aromatherapy with ginger essential oil and the mixture of mint, ginger, and cardamom essential oils were effective in reducing the severity of nausea. No significant reduction in the severity of nausea was found in any of the control and isopropyl alcohol groups [25]. In terms of the effectiveness of aromatherapy with peppermint essential oil, our results were consistent with those of Hunt et al.‘s study. However, in that study, it is unclear which essential oil in the group using the mixture of mint, ginger, and cardamom essential oils caused the reduction in nausea, but in our study, only one essential oil was used in each of the intervention groups. In the study of Zorba et al. (2018), the effects of the two methods of inhalation aromatherapy and aromatherapy with massage on the severity of nausea were investigated in breast cancer patients. In each of the groups, a mixture of 2% peppermint essential oil, 1% bergamot, and 1% cardamom was used. The results showed that inhalation aromatherapy and aromatherapy with massage were equally effective in reducing the severity of nausea [19]. Our results are in line with this study, although it is not clear in Zobra's study that the reduction in nausea severity was related to which of the essential oils in the inhalation aromatherapy groups. In a clinical trial, Joulaeerad et al. (2018) investigated the effect of inhalation aromatherapy with peppermint essential oil on the severity of nausea in 56 pregnant women. The intervention group received 10% peppermint essential oil and the control group received sweet almond oil. Results showed that the mean severity of nausea before and after the intervention was significantly different in each of the study groups, but there was no significant difference between the intervention and control groups [29]. In terms of the effectiveness of aromatherapy with peppermint essential oil, our results were in line with those of Joulaeerad et al. (2018). The use of 10% peppermint oil can be one of the causes of the similarity of results. Evidence suggests that aromatherapy with peppermint essential oil alleviates nausea in three different manners, namely, blocking the serotonin and dopamine receptors, blocking calcium channels and smooth muscle relaxation, and having a direct effect on gastric sphincters [14, 30].

Despite the efficacy of aromatherapy with peppermint essential oil in reducing the severity of nausea in surgical patients, results of some studies indicate that this essential oil does not affect the severity of nausea. Ferruggiari et al. (2012) investigated the effect of inhalation aromatherapy with peppermint essential oil on the severity of postoperative nausea in 70 women. Samples were randomly assigned to one of the three groups of aromatherapy with mint, routine care group, and control group. The results showed no significant difference in the mean severity of nausea before and after the intervention in each study group [21]. Our results are different from these findings. Possible causes of this discrepancy may be differences in study design, peppermint essential oil concentration, limited sample size in the study by Ferruggiari, and the individual characteristics of the study samples.

In a clinical trial, Pasha et al. (2012) investigated the effect of inhalation aromatherapy with peppermint essential oil on the severity of nausea in 60 pregnant women. Samples were randomly assigned to intervention and control groups. The results showed that, in both groups, the severity of nausea had decreased, which the reduction was not statistically significant [22]. Our results are not in line with this study's findings. This discrepancy may be due to differences in study design, duration of intervention, and individual characteristics of the study samples. On the other hand, in the study by Pasha, the concentration of peppermint was not mentioned and the severity of nausea was measured only after the intervention, while in our study, the concentration of peppermint was specified and the severity of nausea was measured before and after the intervention.

In the current study, different types of abdominal surgeries were studied which can be considered as a limitation due to the influence of the extent and location of surgery on nausea. In the present study, colored distilled water was used to blind the subjects in the control group. Also, the distance between the beds in the intervention and control groups was more than two meters. However, it was possible for the peppermint to spread in the environment.

## 5. Conclusion

Our results indicate the equal effectiveness of inhalation aromatherapy with 10% and 30% peppermint essential oils in reducing the severity of nausea in abdominal surgery patients. Due to the ease of use of inhalation aromatherapy, this method is recommended in patients undergoing abdominal surgery. In future studies, it is recommended to investigate the effect of inhalation aromatherapy on only one type of abdominal surgery. It is also suggested to compare the effect of peppermint essential oil with other essential oils such as cardamom and bergamot.

## Figures and Tables

**Table 1:** Demographic characteristics of subjects in study groups (*N* = 120).

Variables		Groups			Test results
		10% peppermint, number (%)	30% peppermint, number (%)	Control, number (%)	
Age (years)	15–30	6 (15.0)	6 (15.0)	9 (22.5)	*F* = 1.10^a^ *p*=0.335
	31–48	13 (32.5)	18 (45.0)	14 (35.0)	
	49–65	21 (52.5)	16 (40.0)	17 (42.5)	
Sex	Female	24 (60.0)	15 (37.5)	22 (55.0)	*X* ^2^ = 4.46 *p*=0.107
	Male	16 (40.0)	25 (62.5)	18 (45.0)	
Type of surgery	Cholecystectomy (laparoscopy)	17 (42.5)	15 (37.5)	14 (35.0)	*X* ^2^ = 6.13 *p*=0.804
	Cholecystectomy (laparotomy)	15 (37.5)	12 (30.0)	11 (27.5)	
	Peritonitis	2 (5.0)	3 (7.5)	2 (5.0)	
	Ileus	4 (10.0)	5 (12.5)	5 (12.5)	
	Cancer of stomach	2 (5.0)	2 (5.0)	4 (10.0)	
	Cancer of colon	0 (0.0)	3 (7.5)	4 (10.0)	
History of previous disease	Yes	12 (30.0)	21 (52.5)	9 (22.5)	*X* ^2^ = 10.61 *p*=0.225
	No	28 (70.0)	19 (47.5)	31 (77.5)	
Duration of anesthesia (minutes)	30–60	11 (27.5.0)	17 (42.5)	12 (30.0)	*F* = 0.273 *p*=0.761
	61–90	22 (55.0)	14 (35.0)	20 (50.0)	
	91–120	7 (17.5)	9 (22.5)	8 (20.0)	
Duration of recovery (minutes)	20–35	21 (52.5)	18 (45.0)	16 (40.0)	*F* = 0.384 *p*=0.682
	36–50	14 (35.0)	14 (35.0)	15 (37.5)	
	51–65	5 (12.5)	8 (20.0)	9 (22.5)	

^a^based on the one-way analysis of variance (ANOVA).

**Table 2:** Mean of nausea before and after intervention in study groups.

Groups	Before, mean ± SD^a^	After, mean ± SD	Test result
10% peppermint	52.31 ± 13.86	40.50 ± 13.35	*t* ^b^ = 12.30
			*p* < 0.001

30% peppermint	60.25 ± 14.98	39.75 ± 12.38	*t* *=* 14.42
			*p* < 0.001

Control	50.68 ± 17.36	47.78 ± 13.72	*t* = 1.05 NS^c^

Result of the one-way analysis of variance	*F* = 4.37	*F* = 8.49	
	*p*=0.015	*p* < 0.001	

^a^standard deviation. ^b^based on the independent samples *t*-test. ^c^nonsignificant.

**Table 3:** Comparing the mean of nausea before and after intervention in study groups.

	Groups	Mean differences ± SD^a^	Test results
Preintervention	Control vs. 10% peppermint	−1.62 ± 3.22	*t* = 0.503^b^ NS^c^
	Control vs. 30% peppermint	−9.56 ± 3.51	*T* = −2.721 *p*=0.008
	30% peppermint vs. 10% peppermint	−7.93 ± 3.46	*t* = 2.189 NS

Postintervention	Control vs. 10% peppermint	6.63 ± 3.22	*t* = 2.060 *p*=0.043
	Control vs. 30% peppermint	7.38 ± 3.12	*t* = 2.364 *p*=0.021
	30% peppermint vs. 10% peppermint	0.750 ± 2.87	*t* = 0.260 NS

^a^standard deviation. ^b^based on the independent samples *t*-test. ^c^nonsignificant.

## Data Availability

The identified datasets analyzed during the current study are available from the corresponding author on reasonable request.
